# An expanded ecological trait dataset and checklist for subterranean spiders of Macaronesia and continental Europe

**DOI:** 10.1038/s41597-026-07699-x

**Published:** 2026-06-30

**Authors:** Diego Patiño-Sauma, Pedro Cardoso, Pedro Oromí, Paulo A. V. Borges, Adrià Bellvert, Luís Carlos Crespo, Isabel R. Amorim, Karla Tolić, Martina Pavlek, Giuseppe Nicolosi, Nuria Macías-Hernández, Stefano Mammola

**Affiliations:** 1 Department of Animal Biology, Edaphology and Geology, University of La Laguna, La Laguna, 38206 Tenerife, Canary Islands Spain; 2 Centre for Ecology, Evolution and Environmental Changes (cE3c), Global Change and Sustainability Institute (CHANGE), Faculdade de Ciências, Universidade de Lisboa, 1749-016 Lisboa, Portugal; 3 Finnish Museum of Natural History (LUOMUS), University of Helsinki, Pohjoinen Rautatiekatu 13, Helsinki, 00100 Finland; 4 University of Azores, CE3C—Centre for Ecology, Evolution and Environmental Changes, Azorean Biodiversity Group, CHANGE —Global Change and Sustainability Institute, School of Agricultural and Environmental Sciences, Rua Capitão João d’Ávila, Pico da Urze, 9700-042 Angra do Heroísmo, Azores, Portugal; 5 Molecular Ecology Group (MEG), Water Research Institute (CNR-IRSA), National Research Council, Verbania Pallanza, Italy; 6 CE3C—Centre for Ecology, Evolution and Environmental Changes, Azorean Biodiversity Group, CHANGE —Global Change and Sustainability Institute, Rua Capitão João d’Ávila, Pico da Urze, 9700-042 Angra do Heroísmo, Azores, Portugal; 7 Croatian Biospeleological Society, Rooseveltov trg 6, 10000 Zagreb, Croatia; 8 Ruder Boskovic Institute, Bijenicka cesta 54, 10000 Zagreb, Croatia; 9 Department of Biological, Geological and Environmental Sciences, University of Catania, Via A.Longo 19, 95125 Catania, Italy

## Abstract

Caves and other subterranean ecosystems impose highly selective environmental filters, driving the evolution of convergent and specialized traits in subterranean organisms. Here, we present the first comprehensive checklist and trait database for subterranean spiders of Macaronesia, thereby filling a significant knowledge gap relative to continental Europe. We compiled data through direct morphological measurements and literature review, covering 64 morphological and ecological traits for 61 species (14 families) from Macaronesia, along with 66 additional species in continental Europe not included in the previous checklist. After accounting for taxonomic changes, the checklist of European subterranean spiders now lists 637 species, of which 278 are considered obligate subterranean dwellers (troglobionts). We used multidimensional hypervolumes to compare the functional spaces of Europe and Macaronesia and explore some putative eco-evolutionary patterns shaping both assemblies. The expanded trait database is a valuable resource for ecological and conservation research, highlighting the need for continued exploration and protection of subterranean biodiversity, including on oceanic islands.

## Introduction

Subterranean ecosystems—such as caves, aquifers, and fissure systems—are considered harsh environments for organisms to establish in, as they impose strong environmental filters on colonizing taxa from the surface species pool^1^. Subterranean taxa have evolved a wide array of morphological, physiological, and behavioral adaptations to survive the conditions imposed primarily by the absence of sunlight and the resulting scarcity of food resources, which shape the typically small and species-poor biological communities found underground^2,3^. As a result, subterranean communities are typically depauperate in species but are notable for high endemism and unique adaptations^2^.

Spiders are among the most ubiquitous predators in terrestrial ecosystems, playing a key ecological role and delivering multiple ecosystem services^4^. This group has successfully colonized the subterranean domain multiple times throughout their evolutionary history, acting as apex predators in most subterranean food webs^5^. Therefore, their diversity and functional adaptations highlight the ecological significance of spider assemblages in subterranean ecosystems.

Despite advances in continental cave biology, the subterranean fauna of oceanic islands such as those in Macaronesia has historically received less attention. These archipelagos offer a unique opportunity to study evolutionary processes in isolated environments and to fill critical gaps in the biogeography of subterranean arthropods^6–8^. Research on subterranean spiders in Macaronesia gained momentum in the late 20th century. In the Canary Islands, interest in cave spiders increased notably in the early 1980s with the establishment of the Group of Speleological Researchers of Tenerife (GIET - Grupo de Investigaciones Espeleológicas de Tenerife, Spain), which focused on biological analyses of caves and species descriptions^9^. Initial investigations in the Azores began in 1987 through expeditions funded by the National Geographic Society and led by researchers from La Laguna University (Tenerife) and the University of Edinburgh (Scotland, UK), exploring caves on Terceira, Pico, and São Jorge islands. These efforts led to the discovery of the first troglobiont species in the archipelago, the spider *Rugathodes pico* (Merrett & Ashmole, 1989)^10,11^.

In the early 1990s, Jörg Wunderlich conducted an extensive study on Macaronesian spiders. He described numerous species from Madeira, including *Centromerus sexoculatus* (Wunderlich, 1992) and *Centromerus anoculus* (Wunderlich, 1995)—the only two troglobiont spiders currently reported from the archipelago—and as many as 19 cave-dwelling species from the Canary Islands^12–14^. In Cape Verde, extensive cave exploration was carried out between 1997 and 1999 by the Speleo Club of Torres Vedras (ECTV- Espeleo Clube de Torres Vedras, Portugal) and the GIET team, during which two still-undescribed troglobitic spiders belonging to the families Hahniidae and Theridiidae were discovered^15^. This pioneering fieldwork paved the way for further research into the high diversity and endemism of subterranean spiders in Macaronesia, providing valuable insights into evolutionary processes such as speciation and adaptive radiation on oceanic islands^16–18^.

Building on this legacy, we present an updated checklist of subterranean spiders in Macaronesia, complementing the existing checklist and trait database of subterranean spiders in continental Europe^19,20^, and release a new trait database for all species included in the checklist, we followed the recently proposed template of 64 morphological and ecological traits for European cave spiders^20^. Additionally, we take this opportunity to update the European database by including missing species—5 species described since 2023, 3 species previously overlooked, and 58 species from the Dinaric karst currently under description. To illustrate the dataset, we constructed a functional space for subterranean spiders in Macaronesia, mapping the position of species and families within this multidimensional space^21^. We also explore the extent of functional convergence between subterranean spider communities in Macaronesia and continental Europe.

## Methods

### Study area – Macaronesian subterranean habitats

The Macaronesian biogeographical region is located in the northeastern Atlantic Ocean and comprises the Azores, Madeira, Selvagens, Canary Islands, and Cape Verde^22^. These archipelagos share common geological characteristics, having all formed from the accumulation of primarily volcanic materials that emerged between the Tertiary and Quaternary periods. As a result, they lack developed karstic landscapes and, consequently, true caves^7^. In this context, lava tubes represent the majority of macro-caverns and are predominantly found in geologically young areas with recent volcanic activity, such as many of the Azores and Canary Islands^23^.

Other widespread volcanic shallow subterranean habitats include networks of small to medium-sized interconnected voids and spaces within the shallow underground—commonly referred to as the *milieu souterrain superficiel*^24^. The MSS is an important habitat in the Canary Islands due to its extensive distribution across the archipelago^18^. Numerous troglobiont species have been described from this environment since its discovery by the GIET team in the 1980s^25^, especially following the improvement of Juberthie’s MSS pitfall trap by H. López and P. Oromí in 2010^26^.

Pyroclastic deposits are also known to harbour subterranean taxa due to their capacity to retain humidity, even at high altitudes^18,27^. In contrast, deep subterranean habitats such as volcanic pits are less common but tend to reach greater depths and persist longer over geological timescales compared to lava tubes. These habitats are more typical of mature islands^18^.

### Checklist compilation

We compiled a checklist of subterranean spiders in Macaronesia based on knowledge accumulated over more than 40 years of field surveys in caves and other subterranean habitats, complemented with a thorough bibliographic survey. In the checklist, we included species that are described, or under description, focusing exclusively on those that carry out at least part of their life cycle in subterranean ecosystems. Conversely, we excluded “accidental species”^28^, namely surface-dwelling spiders accidentally occurring underground. We classified each species listed in the checklist into two ecological categories:

i) Troglophiles, namely species able to maintain stable subterranean populations or prone to inhabit subterranean habitats, being, however, associated with surface habitats for some biological functions or able to maintain surface populations too; and
ii) Troglobionts, for species strictly bound to subterranean habitats.

We are aware this is a somewhat artificial ecological classification that oversimplifies real cases^29^. The ultimate product is an artificial categorization, which will work well for clear-cut cases but may fall short when the association of a species with the subterranean domain is less strict. Still, we provided it to offer a rough indication of the affinity of species for the subterranean medium and consistency with previous checklists on subterranean spiders^19,20^.

We also took the opportunity to update the checklist and trait database for species in continental Europe, updating taxonomy and incorporating newly described species, previously overlooked species, and those currently under description (the latter are identified up to the genus level [morphospecies] and will have their names updated in future database revisions).

### Trait compilation

We here use the definition of “trait” as in the World Spider Trait database^30,31^, whereby a trait is considered any phenotypic characteristic measured at the individual or species level, including morphological, anatomical, ecological, and behavioral attributes. We gathered 64 morphological and ecological traits for every species and morphospecies included in the checklist. These traits are the same ones used to describe the functional space of European subterranean spiders. A detailed description of each trait and its functional significance can be found in^20^. In brief, we collected morphological traits related to body size and subterranean adaptation, as well as ecological traits such as functional guild, foraging strategy, prey range, and type of habitats occupied within the cave. For each species, we collected traits through both direct measurements and literature data (primarily taxonomic descriptions), using individuals of both sexes when available. All measured specimens are stored at the Department of Zoology of the University of La Laguna DZUL collection (species from the Canary Islands), at the DTP – Dalberto Teixeira Pombo arthropod collection (University of the Azores, Angra do Heroísmo, Portugal) (species from the Azores), and at the Croatian Biospeleological Society Collection in Zagreb (undescribed species from the Dinaric karst). We obtained literature through species-specific online searches in the World Spider Catalog^32^ and Google Scholar.

### Analyses to illustrate the dataset

We conducted all analyses using R version 4.1.0^33^. Following the same approach as in^20^, we generated a visual representation of the trait space for subterranean spiders, mapping the position of each species and family from Macaronesia and continental Europe. First, we selected a subset of traits from the complete trait matrix, representing: (i) Species body size and overall morphology (Average body size, Sexual size dimorphism, and Prosoma shape); (ii) Morphological adaptation to the subterranean conditions, including binary (Presence/absence of Eyes, Eye regression), continuous (Femur elongation, Profile reduction, Anterior median eyes [AME], Anterior lateral eyes [ALE], Posterior median eyes [PME], and Posterior lateral eyes [PLE]), ordinal (Pigment), and categorical (AME type) traits; and (iii) Hunting strategy (all binary variables referring to diet, hunting strategy, and food specialization). Then, we carried out data exploration on this trait matrix following^34^. As a result of data exploration, we log-transformed all continuous traits to homogenize their distributions. We also standardized all traits to mean = 0 and standard deviation = 1 to ensure comparable ranges among traits.

Since the trait matrix is a mixture of continuous, binary, ordinal, and categorical traits, we estimated functional dissimilarity among species with a Gower distance^35^. In calculating Gower distance, we attributed equal weight to traits encompassing similar functions within the three groups of traits defined above. This approach resulted in a high-quality hyperspace, with limited distortion of original functional distances among species [quality of 0.99 based on the approach by^36^]. We visualized the trait space as the first two axes of a Principal Coordinate Analysis using the trait dissimilarity matrix as input data. For graphical visualization, we estimated the density of species on the ordination diagram with a kernel density.

Finally, we tested for convergence in the functional space of subterranean spiders in Macaronesia versus continental Europe. For this, we used the three axes of the Principal Coordinate Analysis to construct n-dimensional hypervolumes (*sensu*^37^) for the two pools of species. We computed hypervolumes using a Gaussian kernel density estimator and a default bandwidth for each axis^38^, as implemented in the function *hypervolume_gaussian* in the package ‘hypervolume’ version 3.0.1^39^. We characterized the estimated hypervolumes with functions from the R package ‘BAT’ version 2.7.1^40,41^, calculating their dispersion and regularity^42^. We measured the dissimilarity between the two hypervolumes using the *kernel.beta* function^42^. This estimation decomposes the two processes underlying overall dissimilarity (β_total) among hypervolumes^43^: the replacement of trait space between hypervolumes (β_replacement), and the net differences between the amount of trait space enclosed by the two communities (β_richness).

## Data Records

**Fig. 1:**
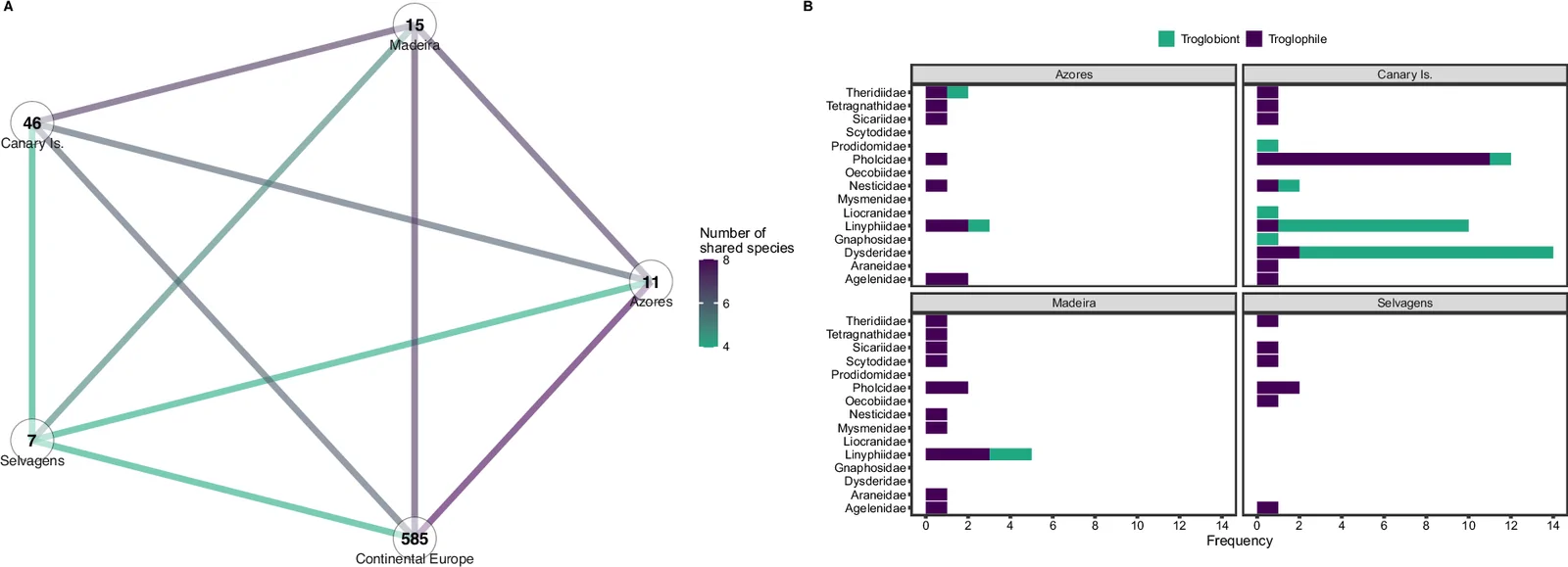
Macaronesian subterranean spiders by numbers. (**A**) Network diagram showing the number of shared species among Macaronesian islands and between the islands and continental Europe. The number inside each dot represents the total number of species on each island and in continental Europe. (**B**) Breakdown of species numbers by archipelago and spider family.

The checklist and trait dataset are available in Figshare^44^ as a tab-delimited file (.csv) and in Excel format (.xlsx). We refer to^20^ for a lengthy description of each trait and its functional meaning. The checklist of Macaronesian subterranean spiders includes 61 species—of which 3 are currently under description—belonging to 14 families (Fig. 1). The dataset also updates the list of species (and trait information) for spiders in continental Europe, adding 77 new species to the list, of which 61 are undescribed species from the Dinaric karst—one of the largest karst landscapes in the world and the largest hotspot of subterranean biodiversity globally^45^. We also made several taxonomic changes and removed some doubtful species. The new checklist (including Macaronesian species) now lists 637 species, of which 278 are considered troglobionts.

## Data Overview

### Macaronesian subterranean spiders by numbers

The most species-rich family is Linyphiidae, with 15 species shared among different genera, especially *Lepthyphantes* (four spp.), *Centromerus* (three spp.), and *Troglohyphantes* (three spp.). Note, however, that preliminary genetic data place the Canarian *Troglohyphantes* in an unrelated Linyphiidae lineage, indicating they probably belong to a separate, still undescribed genus. In second place, Dysderidae is represented by 14 species, all within the genus *Dysdera*, which underwent a large adaptive radiation, especially in the Canary Islands^16,46^. Pholcidae is the third most species-rich family (13 species, 3 genera), with the majority of species belonging to the genus *Spermophorides* (6 spp.) and *Pholcus* (6 spp.), and a single species from the monospecific genus *Ossinissa*. All other families consist of fewer than three species. Linyphiidae and Dysderidae comprise, for the most part, specialised species only found in subterranean habitats and showing traits such as the loss of eyes, appendage elongation, and depigmentation^16^, whereas many Pholcidae are generalist species exhibiting a low degree of morphological specialisation to subterranean life^47^. Concerning the distribution by island, subterranean Dysderidae species seem to occur exclusively in the Canary Islands, whereas other species-rich families such as Pholcidae and Linyphiidae are represented to different extents in almost all archipelagos. The Selvagens Islands and Cape Verde exhibit much lower family and species richness of subterranean spiders—there are still no records of cave spiders in Cape Verde—compared to other islands (Fig. 1B), which may be partly due to research bias, as both archipelagos remain poorly studied. In the case of the Selvagens Islands, another contributing factor could be their small geographic area and the resulting limited availability of subterranean habitats. The islands experience high erosion and have unfavorable conditions for supporting troglobitic fauna, with only one cave known on Selvagem Grande^7^, and two known troglophile spiders. In contrast, Cape Verde is the second-largest archipelago in terms of surface area (4,033 km²) and contains multiple caves, but in general is poorly explored.

Another factor driving cave spider diversity in Macaronesia could be the degree of island nature preservation and habitat intactness. For instance, family and species richness is slightly higher in Madeira compared to the Azores (Fig. 1B), even though the surface area of the latter is almost three times larger, and the Azores has a greater number of caves^48^ and therefore more habitat availability^23^, which can be positively correlated with cave arthropod diversity^8^. However, many of these cavities in the Azores are located in areas heavily impacted by human activity and land-use change. Although a new legal framework for the conservation of Azorean caves has recently been established (see the Regional Legislative Decree No. 10/2019/A of 22 May; Resolution of the Regional Government Council No. 163/2024 of 4 November 2024), these subterranean habitats have historically received limited attention in conservation strategies, and ongoing efforts are needed to ensure their effective protection^8^.

### Comparison between subterranean spiders in Macaronesia and continental Europe

**Fig. 2:**
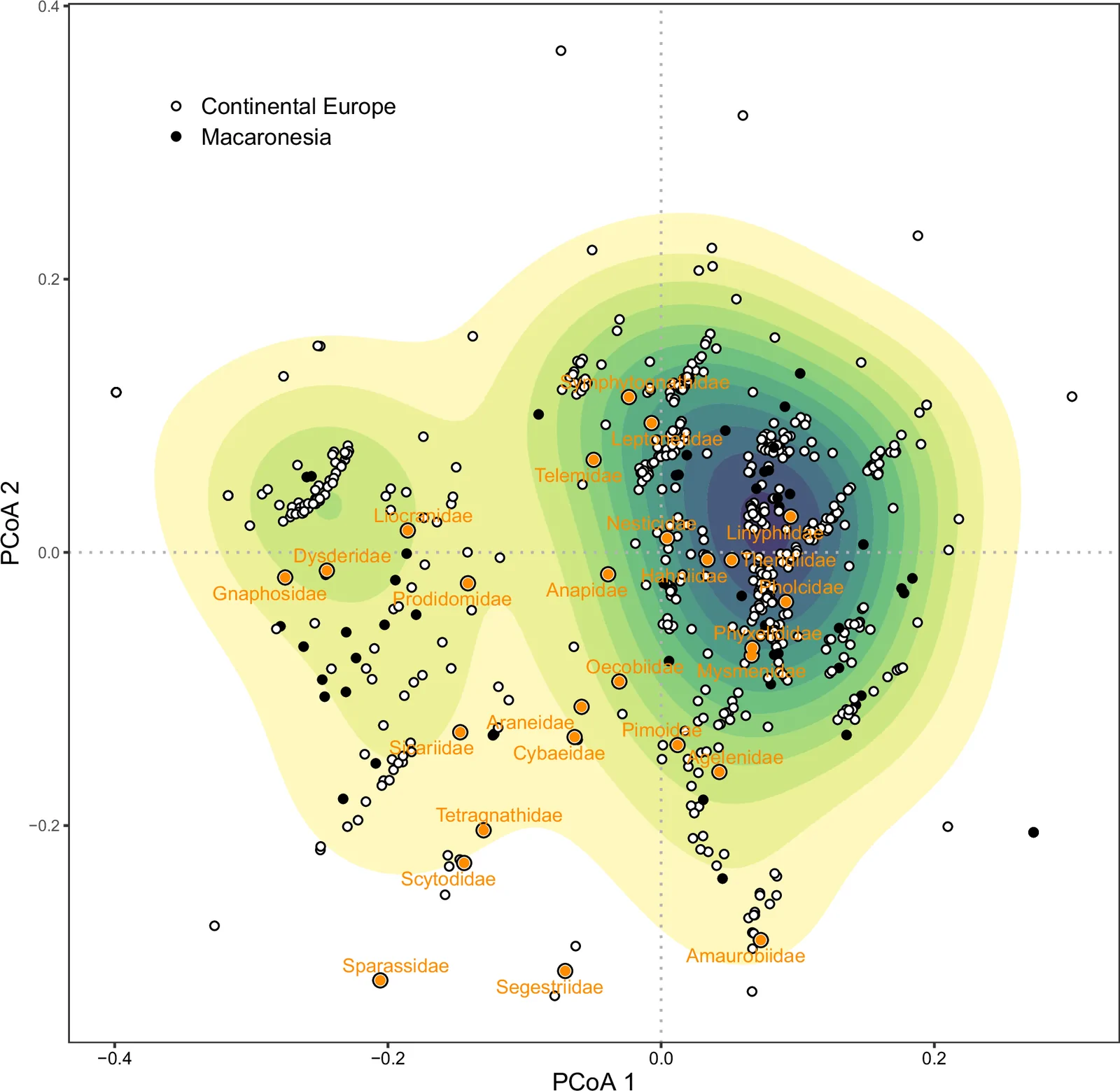
Trait space representation for Macaronesian subterranean spiders. The plot is based on the first two axes of a principal coordinate analysis (PCoA 1 & 2) describing the trait similarity among species. Small black and white dots are individual species, and large orange dots are the centroids for each family. Colour gradient reflects the density of species (higher density in darker areas) and, in turn, highly represented trait combinations.

Using a selection of mostly complete traits from the trait matrix, we mapped the position of each species and family in the trait space (Fig. 2), obtaining a quantification of the functional spread of this trait space and its redundancy (e.g., if multiple species fall within highly sampled areas of the trait space). The overall organization of the trait space is similar to that of the trait space for European cave spiders^20^. Several families are isolated in specific regions of the trait space, suggesting the expression of unique combinations of functions and ecological strategies.

**Fig. 3:**
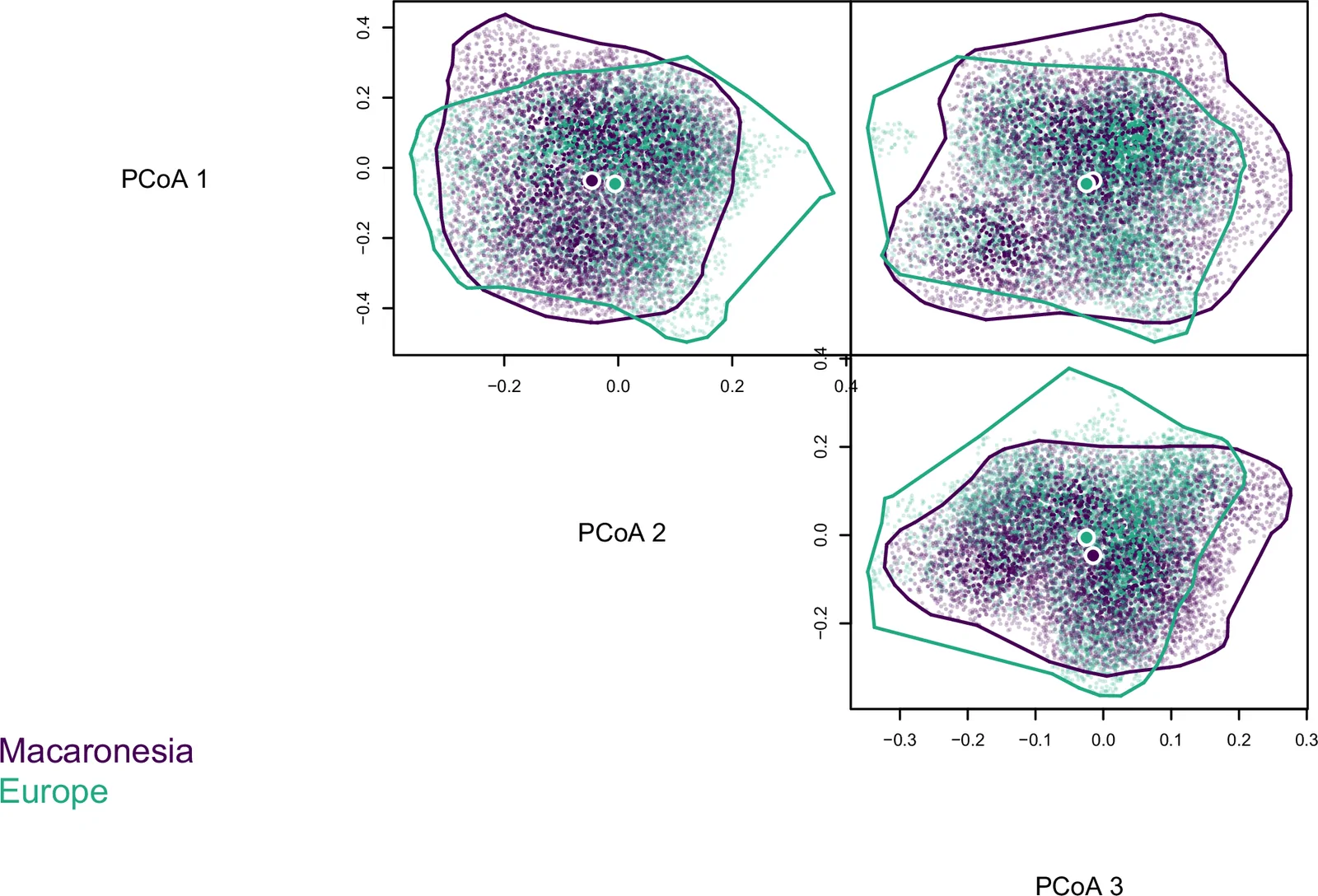
A comparison of the functional space of subterranean spiders in continental Europe versus Macaronesia. For each pair plot, 6000 random points sampled from the estimated 3-dimensional kernel density hypervolumes are shown and represent the real boundaries of the two hypervolumes. Contour lines are drawn only for visual presentation, using the “alphahull” method. Larger dots represent hypervolume centroids. PCoA refers to the axes extracted from the principal coordinate analysis (Fig. 2).

Hypervolume analysis revealed that the functional space of continental European and Macaronesian subterranean spiders moderately overlapped in a multidimensional space (β_total = 0.47; Fig. 3). This degree of overlap suggests an effect of environmental filtering in shaping subterranean ecosystems, leading to some general convergence in the functionality of spider communities in these environments^49,50^. Differences between the two functional spaces were primarily driven by net differences in the amount of trait space enclosed by the two hypervolumes (β_richness = 0.30), rather than by functional replacement (β_replacement = 0.17). This dominance of β_richness in shaping the pattern indicates that, although trait profiles are similar between Macaronesia and continental Europe, the frequency of the most abundant traits differs substantially.

The functional space of subterranean spiders in Macaronesia was more even than that of continental Europe (regularity: 0.28 vs. 0.14), possibly due to lower redundancy in species functions and greater filling of functional niches^51^ in the former. Both trait spaces, however, were similarly dispersed (dispersion: 0.34 vs. 0.31), suggesting that the broader space of functionalities has been similarly explored in both regions.

The specific drivers underlying these patterns are likely multifaceted and may relate to taxonomic similarities and differences between the two species pools, as well as other factors. First, 9 of the 61 species listed in the checklist for the Macaronesian region are also found in continental European subterranean habitats, contributing to some of the observed overlap in functional space (Fig. 1A). These species occur in circum-Mediterranean countries and typically inhabit the twilight zones of caves (e.g., *Loxosceles rufescens*, *Metellina merianae*, *Steatoda grossa*, and two species of *Tegenaria*), as well as the alien species *Eidmannella pallida*, which can likely exploit caves opportunistically across different climatic regions^52^.

Second, it appears that the two regions have undergone somewhat different evolutionary radiations. In continental European caves, the most significant radiation involves Linyphiidae—especially the highly speciose genus *Troglohyphantes*^53,54^—along with Nesticidae and Leptonetidae^19,20^. In contrast, although Linyphiidae are also speciose in the Canary Islands, there has been a major radiation of *Dysdera* in this archipelago, which contributes unique functionalities that are scarcely present in continental Europe—with the notable exception of the Dinaric karst, where other genera of Dysderidae are highly diversified^55,56^.

Third, it is also important to note that our analysis compares the pool of species of Macaronesia and continental Europe, which may be somewhat artificial from a biogeographic standpoint. While continental Europe (and in particular the Iberian Peninsula) is likely the most appropriate reference pool for the Azores and Madeira—the biogeographical context is more complex for the Canary Islands (see^51^). Although the Iberian Peninsula seems to be the most likely source of forest fauna, both due to patterns of wind dispersal colonization^57^ and the presence of paleo-islands until the end of the last glacial maximum^58^, alternative routes of colonization cannot be ruled out for lowland dry areas. Some species from the Canary Islands may, in fact, be more closely related to North African fauna, particularly that of Morocco (e.g.^59–62^). However, the current scarcity of data on subterranean spiders from this region limits further analysis—an issue that could be addressed as soon as new data becomes available.

## Technical Validation

We refer the reader to the former trait dataset description (see^20^) for a full account of technical limitations and data validation steps. However, we report here two main limitations to consider when working with this dataset:

i) Because cave specimens are often scarce—many species were collected only when first described and have not been found since—the dataset combines direct measurements with information extracted from the literature. Unfortunately, many of these descriptions, whether recent or historical, provide limited detail, which restricts the traits that can be reliably extracted (see “Usage Notes” for suggestions on how to handle missing data).
ii) Owing to the dataset’s breadth and the general lack of multiple specimens per species, information on intraspecific variation is also largely absent—except for minimum and maximum body size. Since intraspecific trait variation is known to play a significant role in community ecology^63^ analyses based on this dataset should be interpreted as reflecting average trait values, and results should be contextualized with this limitation in mind^20^.

## Usage Notes

The database is fully compatible with the community composition data for European cave spiders^64^ and other sources of cave-spider-related information. Traits can be uniquely linked to each species included in these datasets using the species’ Latin names. However, taxonomy may need to be checked and updated for some species to account for constant nomenclature changes. This can be done automatically using the R package arakno^65^, which identifies nomenclature changes, synonyms, and spelling errors by querying the World Spider Catalog^32^.

It is worth noting that several traits contain a substantial amount of missing information. As a result, it may be advisable to restrict community-level analyses to traits with good data coverage and work with a reduced matrix including only those well-sampled traits. There are statistical approaches that can partially address gaps in the data. Various imputation techniques are available to estimate missing trait values^66,67^, many of which are implemented in ccthe *fill* function of the R package BAT^40,41^. In addition, community-level analyses can rely on functional distance metrics that tolerate missing entries, such as Gower distances^35^.

### Dedication

We would like to devote this article to Heriberto López, who sadly passed away at the age of 51. He leaves a profound void in the scientific community. He was an enthusiastic researcher who conducted extensive work in the subterranean environment of the Canary Islands. His valuable work and dedication will always be remembered by his colleagues.

## Data Availability

The trait database is available in Figshare^44^ as a tab-delimited file (.csv) and in Excel format (.xlsx). We also deposited traits in the World Spider Trait database (https://spidertraits.sci.muni.cz/^31^).

## Code availability

R code to reproduce the analyses is available on GitHub (https://github.com/StefanoMammola/Cave_Spider_Macaronesia).
